# P-move: a randomized control trial of exercise in patients with advanced pancreatic or biliary tract cancer (aPBC) receiving beyond first-line chemotherapy

**DOI:** 10.1007/s00520-024-08650-9

**Published:** 2024-06-15

**Authors:** Nico De Lazzari, Miriam Götte, Stefan Kasper, Eileen Meier, Martin Schuler, Michael Pogorzelski, Jens T. Siveke, Mitra Tewes

**Affiliations:** 1 Department of Medical Oncology, West German Cancer Center, University Hospital Essen, 45147 Essen, Germany; 2 West German Cancer Center, University Hospital Essen, University of Duisburg-Essen, Essen, Germany; 3 Division of Solid Tumor Translational Oncology, German Cancer Consortium (DKTK Partner Site Essen) and German Cancer Research Center, DKFZ, Heidelberg, Germany; 4 German Cancer Consortium (DKTK), partner site Essen, a partnership between German Cancer Research Center (DKFZ) and University Hospital Essen, 45147 Essen, Germany; 5 Bridge Institute of Experimental Tumor Therapy (BIT) and Division of Solid Tumor Translational Oncology (DKTK), West German Cancer Center, University Hospital Essen, University of Duisburg-Essen, 45147 Essen, Germany; 6 Department of Palliative Medicine, West German Cancer Center, University Hospital Essen, Margot-von-Bonin-Haus, 2. Floor, Room 2.017, Hohlweg 8, 45147 Essen, Germany; 7 National Center for Tumor Diseases (NCT) West, Campus Essen, University Hospital Essen, 45147 Essen, Germany

**Keywords:** Physical activity, Pancreatobiliary cancer, Strength training, Cancer cachexia, Palliative chemotherapy

## Abstract

**Purpose:**

Patients with advanced pancreatic and biliary tract cancer (aPBC) frequently suffer from high symptom burden. Exercise can reduce treatment side effects and improve patient-related outcomes (PROMs). However, evidence from prospective studies regarding feasibility and efficacy in advanced settings are sparse. The primary aim of this prospective, randomized-controlled study was to evaluate the feasibility and effects of exercise (ET) in patients with aPBC.

**Methods:**

Patients with aPBC beyond first-line therapy were randomized according to the minimization procedure with stratification by gender, age, and loss of body weight in the past six months. The intervention group (IG) completed 3 training units/week for 8 weeks (1x supervised strength sessions, 2x individualized home-based sessions). Control group (CG) received recommendations on physical activity during cancer.

**Results:**

41 patients (stage IV pancreatic or biliary tract cancer) were included no adverse events related to exercise occurred during the trial. Physical function increased significantly in IG in 5 out of 7 physical domains. Comparison of IG and CG at 8 weeks (t2) showed significant differences in favour of IG in leg press (*p*=0.001), bench press (*p*=0.011), sit-to-stand (*p*=0.001) and crunch (0.006). Constipation revealed a significant difference in favour of IG at t2 (*p*=0.033). Quality of life stabilized/increased in IG during the study period compared to a decrease in CG. Throughout/Over the 8 weeks, fatigue notably reduced in the IG (*p*=0.028).

**Conclusion:**

Exercise is safe and feasible in patients with aPBC undergoing further line therapy. Significant improvements in physical functioning and increased quality of life were achieved.

German Clinical Trials Register ID: DRKS00021179; Registration date 15.05.2020

**Supplementary Information:**

The online version contains supplementary material available at 10.1007/s00520-024-08650-9.

## Introduction

Advanced pancreatic ductal adenocarcinoma (PDAC) and biliary tract cancer (BTC) exhibit poor overall survival and low 5-year survival rates [1, 2]. Collectively referred to as advanced pancreatobiliary cancers (aPBC), both entities are typically diagnosed at advanced stages with limited surgical options. Despite growing molecular insights and systemic therapies, (multi-)chemotherapy remains the primary approach [3–6]. These patients often bear a substantial symptom burden, including fatigue [7, 8], muscle weakness [9], nausea, anxiety [10], depression, and tumor-associated cachexia [11], affecting nearly 80% of cases. Cancer cachexia, a metabolic syndrome common in aPBC patients, is associated with muscle wasting, fatigue, and non-reversible weight loss despite conventional treatments [12]. Its mechanisms involve altered cytokine levels, inflammation, oxidative stress, and metabolism changes [13], contributing to diminished quality of life and survival [14–16]. In cancer care, exercise has shown promise, reducing fatigue [17], mitigating depression, anxiety, and sleep issues [18], and potentially enhancing quality of life [19]. Beneficial effects on body composition are seen, as well as potential anti-inflammatory effects against cancer cachexia [20]. However, exercise's feasibility and efficacy beyond first-line treatment in aPBC is underexplored, with limited prospective evidence [21–23], lacking clarity on optimal types, doses, and timing, considering the heterogeneity of this population. The P-move study aimed to assess feasibility of exercise beyond first-line chemotherapy in patients with aPBC, evaluating effects on physical function and patient-reported outcomes.

## Materials and methods

### Trial design

P-move was a single-center randomized trial. Patients were randomly assigned to the intervention group (IG), receiving supervised and unsupervised strength training for 8 weeks, or the control group (CG), receiving standard care.

### Recruitment and assignment

Forty-one patients receiving palliative oncological treatment were enrolled from July 2020 to January 2023. Inclusion criteria were age ≥ 18 years, diagnose of advanced pancreatobiliary carcinomas (aPBC) (Stage III-IV), switching beyond first-line chemotherapy after progression of disease, life expectancy of at least 3 months, Eastern Cooperative Oncology Group (ECOG) status ≤ 2 and absence of serious comorbidities contraindicating exercise, such as severe cardiopulmonary disease, (EF<45%, heart failure NYHA III-IV, severe respiratory partial or global failure, uncontrolled hypertension), uncontrolled central nervous system metastases or bone metastases with risk of pathologic fracture (trial protocol DRKS00021179). After giving informed consent, each patient underwent baseline assessments prior to randomization using the principle of minimization, considering age, sex, and recent weight loss. Personal and medical data were pseudonymized with identification codes. Blinding of participants and scientist wasn't feasible due to the intervention's nature.

### Equity, diversity, and inclusion statement

The author group consists of male and female investigators from different disciplines. Our study population included both male and female subjects with aPDAC or aBTC.

### Intervention

The intervention consisted of 3 exercise sessions per week for a duration of 8 weeks. One training session per week was delivered supervised by a qualified exercise physiologist and 2 training sessions were unsupervised at home. The focus of the intervention was resistance exercise targeting the main muscle groups. Each supervised exercise session consisted of a 5-minute warm-up on a bicycle ergometer and 40 minutes of resistance exercise focused on hypertrophy. Supervised resistance training consisted of the following exercises: leg press, bench press, latissimus pulldown, crunch and back extension. Each exercise contained 3 sets of twelve repetitions with two-minute breaks between sets. The initial load of resistance was determined using the hypothetical one-repetition maximum (h-1RM) regarding the Brzycki formula [24]. All participants started with 50% of the h-1RM to ensure safety. Progressions during the 8-week period were applied depending on the participants’ feedback and performance the week before. Since our patient group was fragile due to the tumor burden, a reduction in exercise load was necessary if patients reported fatigue or muscle soreness.

Home-based exercise consisted of bodyweight and resistance band exercises tailored to individual needs, with an included resistance band (Supplementary file 1).

### Usual care/control group

The CG attended a single exercise counselling session, offering general guidance on physical activity during cancer. Recommendations aligned with current scientific insights [25, 26] (Supplementary file 2).

### Primary outcomes

P-move’s primary aim was to evaluate the feasibility of tailored strength exercises beyond first-line treatment for aPBC patients. This encompassed recruitment and dropout rates, adherence to both supervised and home-based sessions, and exercise safety. All withdrawals were considered as drop-outs, with reasons noted. The exercise physiologist monitored adherence to the supervised sessions. For home-based exercise, patients reported completion frequency weekly during supervised sessions. Adverse events (related to therapy or exercise) were evaluated using Common Terminology Criteria for Adverse Events (CTCAE) Version 5.0.

### Secondary outcomes

Secondary outcomes were differences in physical function (h-1RM of leg press, bench press, crunch, back extension, latissimus pulldown, handgrip strength and 1-minute-Sit-to-Stand (1-m-STST) within groups and between groups measured at baseline (t0), after 4 weeks (t1) and after 8 weeks (t2). Furthermore, we evaluated the differences within and between groups in quality of life with the European Organization for Research and Treatment of Cancer Quality of Life Questionnaire (EORTC-QLQ-C30) and Physical Activity, Exercise, and Sport Questionnaire (BSA) [27, 28].

### Statistical analysis

The trial’s pilot nature prompted us to estimate sample size following Kieser and Wassmer’s rules of thumb [29] for two-armed pilot trials. The primary endpoint assessed exercise feasibility beyond first-line treatment, encompassing recruitment, adherence, drop-outs, and safety. Descriptive statistics included all participants. Baseline characteristics were presented means, medians, ranges (quantitative variables), and numbers/percentages (categorical variables). Non-parametric tests (Mann-Whitney U Test and Wilcoxon rank-sum test) utilized for intergroup and intragroup outcomes. Statistical analysis was performed using SPSS version 29 (IBM, USA) with an intention-to-treat approach. Significance was set at *p* < 0.05 (2-tailed) across all tests.

### Registrations and approvals

This study was performed in line with the principles of the Declaration of Helsinki. The Ethics Committee of the Medical Faculty University of Duisburg-Essen approved this study prior to participant inclusion (19-8843-BO).

## Results

### Recruitment

**Fig. 1:**
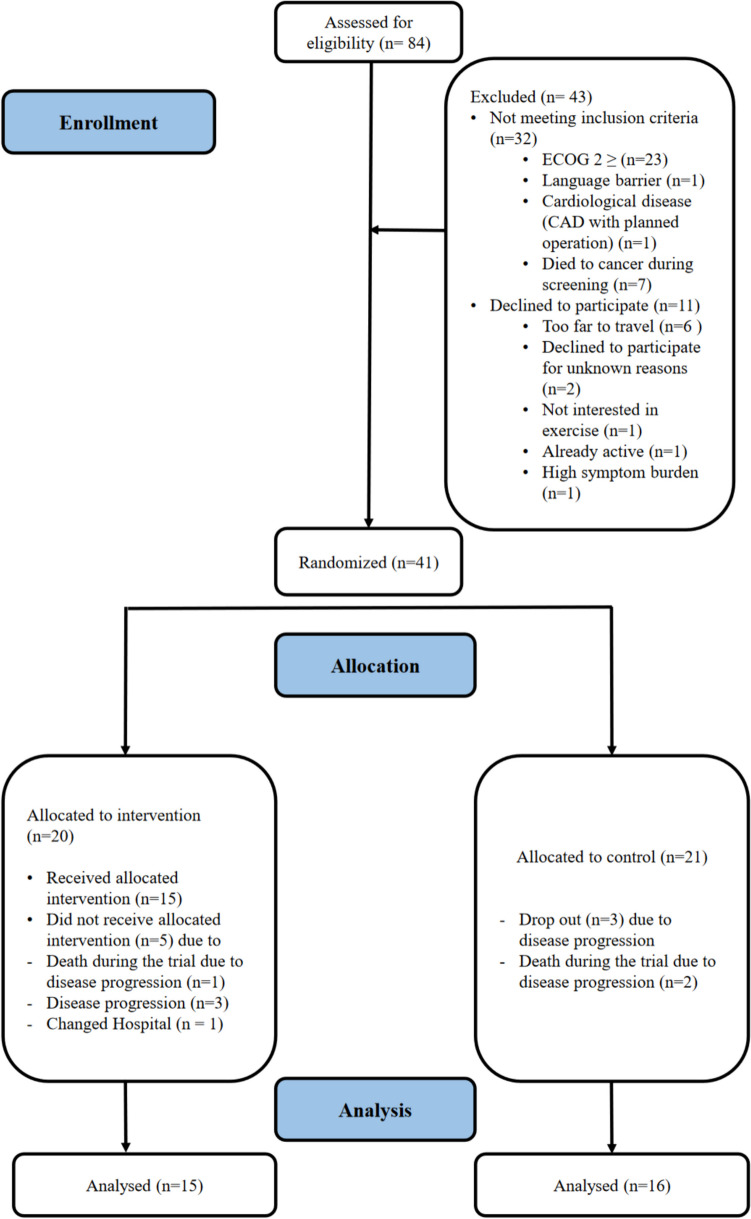
CONSORT flow diagram

From July 2020 to January 2023, 84 patients from the West German Cancer Center were screened whereof 52 (61%) fulfilled inclusion criteria. In total, 41 patients (78%) participated as described in Fig. 1.

P-move participants exclusively had stage IV cancer (100%). They averaged 58.7 years (SD 12.08) with a normal BMI of 23.7 (SD 4.7). Patients had received an average of 14.58 chemotherapy cycles (SD 11.08) over 2.5 treatment lines (SD 1.0) with enrolment. We enrolled 16 patients (39%) with over 5% weight loss in the last 6 months, constituting the cachexia group. Table 1 outlines the baseline characteristics by group.

**Table 1:** Baseline characteristics of study population

	Groups		
	Control group (*n*=16)	Intervention group (*n*=15)	Drop-out group (*n*=9; 4 IG/5 CG)*
Anthropometric data			
Sex, n (male/female)	8/8	6/9	4/5
Age, in years Median (Range)	63.0 (37-74)	61 (30-86)	58.0 (51-72)
BMI; Median (Range)	24.4 (17.1-37.4)	22.1 (17.4-32.6)	19.8 (15.7-26.0)
Disease specific data			
Tumor site, n (%)			
biliary tract	6 (37.5%)	6 (40%)	0 (0%)
pancreas	10 (62.5%)	9 (60%)	9 (100%)
Stage, n (%)			
IV	16 (100%)	15 (100%)	9 (100%)
Metastases, n (%)			
Yes	16 (100%)	15 (100%)	9 (100%)
Site of metastases, n (%)			
Liver	10 (62%)	9 (60%)	8 (89%)
Peritoneum	8 (50%)	7 (46%)	4 (56%)
Lung	1 (6%)	1 (7%)	2 (22%)
Bone	0 (0%)	1 (7%)	1 (11%)
Others^a^	4 (25%)	5 (33%)	3 (22%)
Weight loss past 6 months, n (%)			
No weight loss	9 (56%)	5 (33%)	1 (11%)
0-2% weight loss	0 (0%)	3 (20%)	2 (22%)
2-5% weight loss	1 (6%)	2 (13%)	1 (11%)
>5% weight loss	6 (38%)	5 (33%)	5 (56%)
Treatment specific data			
Chemotherapy lines at study entry; Median (Range)	2 (2-6)	2 (2-5)	2 (2-4)
Chemotherapy cycles prior to inclusion; Median (Range)	10 (2-54)	12 (1-34)	17 (5-24)
Starting Chemotherapy at T0			
Gemcitabine based	7 (44%)	9 (60%)	6 (67%)
5-FU based	6 (38%)	6 (40%)	1 (11%)
Nanoliposomal irinotecan based	3 (19%)	0 (%)	2 (22%)
Blood sampling (t0)			
C-reactive protein, mg/dl	2.2. ± 4.6; 0.4 (0-18.3)	0.8 ± 0.8; 0.5 (0-2.2)	9.0 ± 8.8; 5.10 (0-24.2)
Interleukin 6, pg/ml	7.3 ± 8.8; 6.8 (0-30.7)	16.0 ± 34.8; 6.4 (0-140)	51.1 ± 8.8; 25.9 (0-291)
CA 19-9, U/ml	33645.5 ± 7570.4; 294 (0-28684.4)	6763.4.1 ± 21786; 176.7. (0-85139.2)	25334.1 ± 57692.5; 2255.2 (14-176817)
Neutrophil/lymphocyte ratio %	3.53 ± 3.8; 2.1 (0.9-16.1)	2.3 ± 1.2; 2.1 (0.7-5.5)	7.4 ± 4.7; 6.9 (2.3-16.8)

*1 Drop-out of the missing patient (withdrew immediately after inclusion, changed hospital), the patient was not included within the baseline characteristic

^a^Lymph nodes, ovarian

### Feasibility

#### Recruitment rate

Of the 84 aPBC patients screened (Fig. 1: CONSORT Flow Diagram), 32 were ineligible, mostly due to ECOG score ≥ 2 (*n*=23) or death between screening and inclusion (*n*=7). Among the 52 eligible patients, not participating was mainly due to distance from the study center (*n*=6), unexplained refusal (*n*=2), lack of exercise interest (*n*=1), existing physical activity (*n*=1), or excessive symptom burden from antitumor therapy (*n*=1). Overall recruitment reached 78%.

#### Drop-out rate

At the final assessment, 31 out of 41 patients provided complete data, resulting in a dropout rate of 24% (*n*=10). Reasons included disease progression/decline in health (*n*=6), cancer-related death (*n*=3), and a change in treatment hospital (*n*=1). Dropouts were evenly distributed between IG and CG, each with 5 patients, all of whom had PDAC. In comparison between completers and drop-outs, blood sampling at inclusion revealed significantly higher inflammatory parameters (interleukin 6 *U*= 61.50, *Z*=-2.537, *p*=0.011; neutrophil/lymphocyte ratio (NLR) *U*=42.00, *Z*=-3.158, *p*<0.00; C-reactive protein *U*=46.00, *Z*=-3.082, *p*=0.002) in dropouts. Lower metabolic parameters (calcium *U*=77.50, *Z*=-2.01, *p*=0.044), organ function (e.g., liver and thyroid) (alkaline phosphatase *U*=52.50, *Z*=-2.18, *p*=0.005, gamma-glutamyltransferase *U*=47.00, *Z*=-2.996, *p*=0.003, L-lactate dehydrogenase *U*=66.50, *Z*=-2.37, *p*=0.018, albumin *U*=28.00, *Z*=-3.623, *p*<0.001). There were lowered triiodothyronine values (*U*=33.50, *Z*=-0.421, *p*<0.001) and higher thyroxine values (*U*=65.00, *Z*=-2.414, *p*=0.016) at baseline measurement.

#### Adherence to exercise

IG participants completed 75% of scheduled exercise sessions (273/360) across 8 weeks, ranging from eight to twenty-four on an individual basis. Adherence stood at 83% (80/90) for supervised sessions and 71% (172/240) for home-based sessions. Four patients (2 PDAC, 2 BTC) reported <75% training perception, while 11 achieved >75% adherence. Predominantly, negative tumor therapy effects were reasons for non-participation.

#### Adverse events

Table 2 shows the overall adverse events during the trial. The most common adverse events were related to the cytotoxic side effects of antitumor therapy, followed by tumor-related adverse events and infectious disease.

**Table 2:** Adverse events during trial participation

AE grade	Intervention group	Control group
1	***n*= 20** thrombocytopenia (*n*=1), fatigue (*n*=3), pain (*n*=2), fever (*n*=2), neutropenia (*n*=1), nausea (*n*=2) dyspnea (*n*=3), shoulder pain (*n*=1), diarrhea (*n*=1), polyneuropathy (*n*=1), sore throat (*n*=1) obstipation (*n*=1), abdominal pain (*n*=1)	***n*= 21** nausea (*n*=1), fatigue (*n*=2), edema (*n*=3) fever (*n*=1), obstipation (*n*=2), dyspnea (*n*=1), abdominal pain (*n*=2), thrombocytopenia (*n*=2), ascites (*n*=3), mucositis (*n*=2), diarrhea (*n*=1), anemia (*n*=1)
2	***n*= 13** edema (*n*=1), diarrhea (*n*=2), obstipation (*n*=1), fever (*n*=1), conjunctivitis infective (*n*=1), anemia (*n*=2), shoulder inflammation (*n*=1), ascites (*n*=2), fatigue (*n*=1), thrombocytopenia (*n*=1)	***n*= 17** pain (*n*=1), obstipation (*n*=1), neutropenia (*n*=1), nausea (*n*=3), vomit (*n*=1), bronchial infection (*n*=1), dysphagia (*n*=1), thrombocytopenia (*n*=2), fatigue (*n*=2), ascites (*n*=1), anemia (*n*=3)
3	***n*= 1** Portal vein thrombosis (*n*=1)	***n*= 9** port infection (*n*=1), anemia (*n*=2), hypokalemia (*n*=1), neutropenia (*n*=2), ascites (*n*=2), diarrhea (*n*=1)
4	***n*= 5** disease progression (*n*=3) hospitalization due to disease progression (*n*=2)	***n*= 4** pancytopenia (*n*=1), neutropenia (*n*=1) hospitalization due to disease progression (*n*=2)
5	***n*= 1** Cancer related death (*n*=1)	***n*= 2** Cancer related death (*n*=2)

Across the IG, 39 adverse events were observed during the trial, compared to 54 in the CG. Both groups reported 6 adverse events rated as >3 in severity. Three patients entered the study with pre-existing shoulder issues: two with shoulder joint osteoarthritis and 1 with inflammation, resulting in personalized exercise adjustments. The interdisciplinary team deemed the likelihood of adverse events being exercise-related as low.

### Secondary outcomes

#### Physical function

**Fig. 2:**
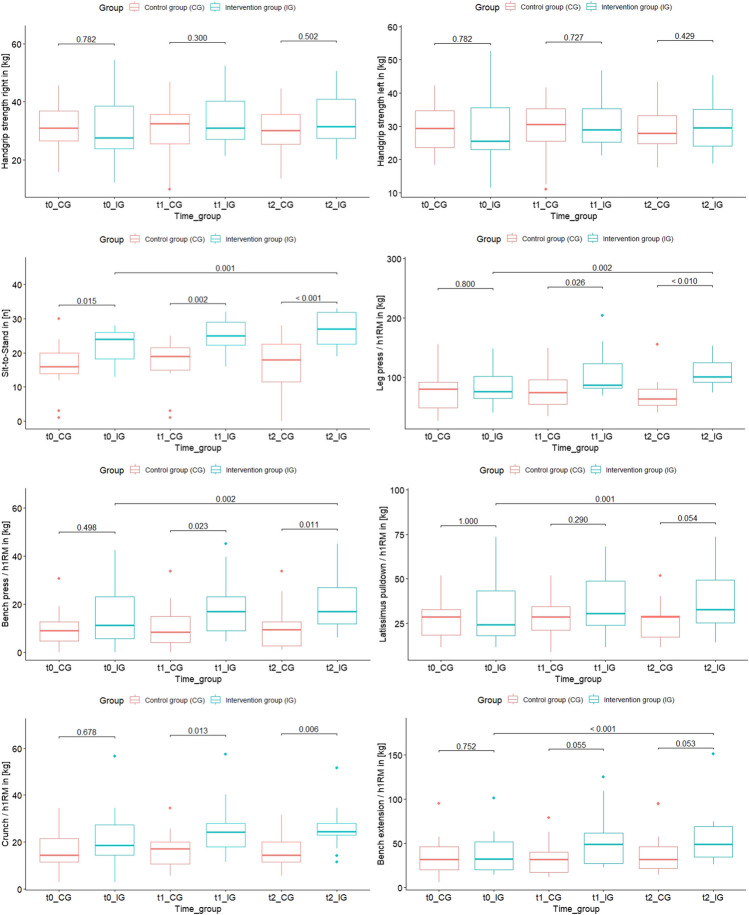
Physical function data at all 3 measurement points (t0,t1,t2) displayed. Significant difference between groups and intragroup differences are shown with *p*-values. Full test statistics are listed in the results text

All physical domains are presented in Fig. 2, and significant differences within and between groups are shown at all measurement points.

#### Handgrip strength

For left handgrip strength, there were no significant intergroup differences at t0 (*U*=113.00, *Z*=-0.277, *p*=0.782), t1 (*U*=103.00, *Z*=-0.087, *p*=0.30), and t2 (*U*=103.00, *Z*=-0.672, *p*=0.502). Likewise, right handgrip strength showed no significant differences: t0 (*U*=113.00, *Z*=-0.277, *p*=0.782), t1 (*U*=97.00, *Z*=-0.349, *p*=0.727), and t2 (*U*=100.00, *Z*=-0.791, *p*=0.429). Delta changes for left handgrip: CG mean -1.00 kg. (CI -3.43 – 1.42), IG mean change 1.31 kg. (CI -1.69 – 4.32). Delta changes for right handgrip: CG mean -0.71 kg. (CI -2.90 – 1.48), IG mean change 1.96 kg. (CI -1.21 – 5.15).

#### One-minute Sit-to-Stand-Test

The functional 1-m-STST showed a significant difference in favour of the IG group at baseline t0 (*U*=58.50, *Z*=-2.437, *p*=0.015), t1 (*U*=35.00, *Z*=-3.062 *p*=0.002) and t2 (*U*=32.50, *Z*= -3,464, *p* < 0.001). Within-group comparisons showed a significant increase in IG (t0 median 24, t2 median 25 *z*= -3.294 *p*=0.001). Delta change of CG mean 0 repetitions confidence interval (CI) -2.54 – 2.54 compared to IG mean change 4.33 repetitions (CI 2.35 – 6.31).

#### Leg press

Baseline measurements showed no significant differences. However, after 4 weeks of exercise, leg press strength significantly favored the intervention group (IG) (*U*=54.00, *Z*=-2.228, *p*=0.026). At the final measurement (t2), IG demonstrated a substantial advantage over the CG (*U*=32.00, *Z*=-3.481, *p*<0.001). These trends were consistent in within-group comparisons, where IG exhibited a significant increase over the 8-week training (t0 median 74.56, t2 median 97.51, *Z*=-3.160, *p*=0.002). Delta changes in leg press CG mean -6.86 kg. (CI -15.92 – 2.20), IG mean change 22.27 kg. (CI 14.37 – 30.17).

#### Bench press

No intergroup difference was noted in baseline measurements (*U*=32.00, *Z*=-0.686, *p*=0.498). However, after 4 weeks of exercise, a significant advantage favored the IG over the CG (*U*=48.00, *Z*=-2.281, *p*=0.023). This pattern persisted at t2, favoring the IG (*U*=50.50, *Z*=-2.558, *p*=0.011). Within the exercise group, a noteworthy increase over time was seen (t0 median 10.63, t2 median 16.31, *Z*=-3.107, *p*=0.002). Delta changes in chest press strength CG mean 0.34 kg. (CI -1.82 – 2.50), IG mean change 5.20 kg. (CI 2.69 – 7.70).

#### Latissimus-Pulldown

There was no significant difference between groups at t0, t1 or t2 for latissimus strength. At t2, the results were close to reveal a significant difference (*U*=71.50, *Z*=-1.919 *p*=0.055). Within-group comparisons showed a significant increase in IG over the study period (t0 median 22.94, t2 median 30.01, *Z*=-3.238 *p*=0.001). Delta change of CG mean -1.41 kg. (CI: -3.04 – 0.22) compared to IG mean change 6.87 kg. (CI: 3.58 – 10.15).

#### Crunch

No intergroup difference was noted in baseline measurements (*U*=109.50 *Z*=-0.416 *p*=0.678). During study participation at t1 (*U*=48.00, *Z*=-2.490, *p*=0.013) and t2 (*U*=50.50, *Z*=-2.750, *p*=0.006), significant changes in favour of IG were observed. Additionally, within-group analysis revealed a near-significant increase from t0 to t2 in the IG (t0 median 17.25, t2 median 23.00, *Z*=-2.731, *p*=0.06). Delta change of CG mean -1.81 kg. (CI: -4.09 – 0.47) compared to IG mean change 5.21 kg. (CI: 1.09 – 9.34).

#### Back extension

No intergroup difference was noted in baseline measurements (*U*=112.00, *Z*=-0.316, *p*=0.752), t1 (*U*=61.00, *Z*=-1.922, *p*=0.055) and t2 (*U*=71.00, *Z*=-1.938, *p*=0.053). Within-group comparisons showed a significant increase within the IG from t0 to t2 (t0 median 31.54, t2 median 46.00, *Z*=-3296, *p*<0.001). Delta change of CG mean 3.40 kg. (CI: -3.21 – 10.02) compared to IG mean change 21.72 kg. (CI: 8.27 – 35.17).

#### Patient-related outcome measurement (PROM)

Table 3 presents patient-related outcomes, assessing quality of life and physical activity levels using validated questionnaires. A significant difference between groups emerged at t2 for constipation (*U*=69.000, *Z*=-2.132, *p*<0.033). Throughout the trial, fatigue notably reduced in the IG (*Z*=-2.199, *p*<0.028). According to the BSA questionnaire, a noteworthy difference in weekly exercise minutes favored IG at t2 (*U*=56.000, *Z*=-2.505, *p*<0.012).

**Table 3:** Patient-related outcomes

*n*=31	Mean CG (SD) t0	Mean CG (SD) t2	Mean IG (SD) t0	Mean IG (SD) t2	Group difference (IG vs. CG) at t2 p-value
European Organization for Research and Treatment of Cancer Quality of Life Questionnaire (EORTC-C30)					
Global health status (QoL)	52.34 (22.15)	50.52 (16.24)	53.61 (21.06)	55.55 (23.91)	0.423
Functional scales					
Physical function	69.17 (19.30)	65.00 (21.15)	67.78 (25.25)	72.00 (28.19)	0.232
Role functioning	55.21 (35.85)#	53.13 (28.69)#	58.33 (35.85)	63.69 (36.92)	0.240
Emotional functioning	45.31 (29.81)#	58.85 (20.74)#	64.07 (21.17)	65.00 (27.67)	0.572
Cognitive functioning	65.62 (31.31)	67.71 (20.61)	70.00 (26.13)	73.33 (23.40)	0.470
Social functioning	45.83 (33.61)	61.46 (28.36)	51.11 (30.52)	56.67 (38.73)	0.861
Symptom scales					
Fatigue	56.94 (29.50)	54.86 (21.26)	59.25 (24.36)	44.44 (30.28)#	0.140
Nausea and vomiting	23.96 (25.07)	17.71 (22.33)	8.34 (8.64)	9.44 (15.71)	0.520
Pain	38.54 (33.73)	21.87 (19.92)#	40.00 (33.73)	23.33 (25.04)	0.953
Dyspnoea	41.67 (31.03)	37.50 (29.50)	42.22 (40.76)	40.00 (40.24)	0.953
Insomnia	54.17 (34.16)	45.83 (31.91)	42.22 (36.66)	33.33 (35.63)	0.232
Appetite loss	31.25 (30.96)	27.08 (32.70)	28.57 (28.82)	26.67 (36.08)	0.922
Constipation	39.58 (36.96)	37.50 (31.91)	20.00 (30.35)	15.56 (21.33)	0.045
Diarrhoea	31.25 (35.42)	35.42 (37.45)	33.33 (37.80)	30.00 (32.2)	0.711
Financial difficulties	33.33 (36.51)	16.67 (24.34)#	31.11 (42.66)	31.11 (44.48)	0.599
Physical Activity, Exercise, and Sport Questionnaire (BSA)					
Movement activity in free time (min/week)	298.91 (446.39)	273.80 (488.76)	356.07 (392.26)	311.83 (339.61)	0.325
Climbing stairs (Floors per day)	12.44 (14.84)	25.08 (36.29)	17.75 (35.03)	19.83 (26.44)	0.870
Exercise or sport activities (min/week)	37.66 (114.69)	36.18 (84.27)	22.45 (69.59)	117.33 (126.17)	0.019
Physical activity at work (min/week)	0.88 (1.5)	1.27 (2.66)	1.20 (1.74)	0.29 (0.83)	0.505

*IG* Intervention group, *CG* Control group

# *p*< .05 significant difference within group from t0 to t2

Subgroup analysis within aPDAC (*n*=19: IG, 9; CG, 10) displayed a significant advantage for IG in the physical function domain of quality of life at t2 (*U*=23.500, *Z*=-1.761, *p*<0.017). In the aBTC subgroup, the exercise group showed notably reduced constipation symptoms at t2. For the highly cachectic subgroup with over 5% body weight loss in the last 6 months (*n*=11: IG, 5; CG, 6), the quality-of-life score favored IG (*U*=1.000, *Z*=-2.586, *p*<0.009), and fatigue was significantly lower in IG (*U*=3.000, *Z*=-2.196, *p*<0.030) than in the CG.

## Discussion

P-move investigated the feasibility and effects of exercise in patients with advanced PDAC or BTC beyond first line systemic treatment. We conducted a randomized controlled trial comparing patients who received exercise with those who were advised to exercise with the aim of assesing the feasibility of exercise based on recruitment rate, drop-out rate, adherence to exercise and adverse events. Within the IG, no adverse events related to exercise were reported, and a combination of supervised and guided home-based exercise during second-line treatment is considered safe. Compared to other trials in advanced pancreatic and biliary tract cancer (aPBC), our recruitment rate was distinctly higher [30, 31]. One reason for this may be that the study was conducted in a single-center comprehensive cancer center with the highest standard of care within a metropolitan area, which offers a shorter distance to the study centre, allowing more patients to participate without significantly increasing travel times. The drop-out rate was high as expected and comparable to other trials with advanced cancer patients [32, 33]. Patients demonstrated high adherence to both supervised and home-based training. Overall, P-move demonstrates the safety of exercise in this highly burdened patient population. We clearly demonstrate that even beyond first-line treatment exercise can affect physical function and improve patients’ well-being.

### Clinical Implications

Based on recent exercise trials [30, 34] in advanced cancers, we expected exercise to provide physical and psychosocial benefits. Given the poor prognosis of PDAC and BTC, as assessed in our study, the treatment's emphasis lies in enhancing or maintaining quality of life, alleviating symptoms, and extending survival time. A general strength of our trial was the clinically embedded and feasible way of delivering (combination of supervised and home-based training) exercise intervention to patients under palliative systemic treatment without being too time consuming. We were able to integrate exercise within the current ambulant treatment system. Mostly, we offered the supervised exercise sessions before receiving anticancer treatment, or patients could decide which day would fit the best. Our results show that exercise can ensure and enhance patient mobility. These results, considering the frequent weight loss and loss of muscle mass during the trajectory of disease are an important signal that physical ability can be maintained or even improved by systematic and regular supervised exercise. These findings are important because cancer cachexia and sarcopenia are common and clinically significant challenges in aPBC, affecting physical functions, quality of life and reducing survival time. Pancreatic cancer patients are known to have physical function levels in the upper and lower body below the health reference values [35]. In particular, the functionality of the lower extremities enables patients to participate actively in social life and to cope optimally with regular everyday life. A qualitative study of advanced tumor patients with cachexia showed that patients want to take advantage of the positive potential of exercise and want to be as fit as possible during their therapy [36]. In our study we recognized that patients receiving exercise had fewer adverse events related to anti-tumor therapy. These findings support the hypothesis of an additional positive effect of exercise. A recent study showed that patients with advanced cancer who exercised during chemotherapy had significantly fewer chemotherapy dose delays, dose reductions [37, 38] or thrombocytopenia [39].

Despite the non-significant difference between groups in most patient-related outcome domains, we demonstrated a stabilization and slight increase in quality of life over 8 weeks of exercise in the intervention group. The EORTC-C30 demonstrates a general trend, with functional scales stabilizing or increasing slightly over the course of the trial and symptom scales decreasing in IG. Our findings gain increased significance based on the physical function scale as a recommended prognostic factor in PDAC [40]. Despite the same development/trend in the CG, the changes are considerably smaller. Subjective physical performance in our control group worsened over the course of study participation incorporate importance of exercise. Here, the change in chemotherapy plus the additional care aspect could be decisive. Quality of Life and physical function slightly decrease during study participation in CG compared to an increase in IG. One of the most common symptoms at the palliative cancer stage is fatigue, which affects almost 80% of all patients with advanced cancer [8]. From t0 to t2, our IG experienced significantly less fatigue. These findings are similar to those of Yeo et al. [41] during adjuvant treatment and highlight the beneficial aspect of exercise in palliative treatment. Based on our survey of patients' physical activity, the IG showed a significantly higher level of sports activity, which has a particular impact on fatigue and underlies the significant difference within group [42, 43].

Given our baseline blood sampling and the noteworthy differences between study completers and drop-outs, future investigations might benefit from using blood sampling as a stratification method during randomization. This approach could help balance dropouts across groups. Given NLR's established prognostic significance in both BTC [44, 45] and PDAC [46], along with its inclusion in routine clinical blood tests, it's prudent to consider it as a potential factor in randomization procedures.

### Limitations

Even though our results demonstrate a benefit of exercise beyond first-line palliative treatment, our small sample size limits its generalizability. Based on the feasibility character of P-move, we did not power the study based on our secondary outcome; therefore, the results should be seen as providing a hypothesis to be tested in another upcoming larger multicentre randomized controlled trial. In P-move, we included 2 tumor types. Despite the limited number of cases, no distinct disparities in feasibility or exercise effects between these entities were identified. Notably, both tumor types exhibited similar responses to exercise. By demonstrating the benefits of exercise in cachectic patients, our study suggests a need for future research concentrating on those with tumor cachexia. Despite the small sample size, significant differences were observed, underscoring the potential impact. A major limitation is the impact of tumor therapy response on our cohort, which significantly influences psychological and physical aspects of patient-reported outcome measures (PROMs), though this variable remains uncontrolled. The method of measuring adherence to home-based training could not be fully tested by solely interviewing patients. Future studies should use validated tools to measure patient activity at home, such as accelerometers, devices such as smartwatches or pedometers, to control for reporting bias.

### Supplementary information

ESM 1 (DOCX 14311 kb)

ESM 2 (DOCX 15 kb)

## Declarations

### Patient and public involvement

Patients and/or the public were not involved in the design, or conduct, or reporting of this research.
