# Supplementary material for: P-move: a randomized control trial of exercise in patients with advanced pancreatic or biliary tract cancer (aPBC) receiving beyond first-line chemotherapy
Source: Support Care Cancer. 2024 Jun 15;32(7):437. doi: 10.1007/s00520-024-08650-9 (PMC11180022; doi:10.1007/s00520-024-08650-9)
Supplement: Supplementary file 1 — (DOCX 14311 kb) [file 520_2024_8650_MOESM1_ESM.docx]

**Home-based Training schedule**

**Exercise catalog Warm-up approx. 5-10 minutes**

**Activation cardiovascular system**

| **Exercise** | **Execution** |
| --- | --- |
| Bicycle ride/stationary or outside | The intensity for the general warm-up should be 30 watts for the first training session. The warm-up should not be exhausting, but only serves to warm up the cardiovascular system. |
| Walking/fast walking /running | As an option to cycling, the warm-up can take place on the treadmill or going for a stroll, activating the cardiovascular system. |

**Exercise catalog (approx. 40 minutes)**

**Take a break for 1-2 minutes between sets and different exercises. In case you need more time to recover from exhaustion increase time between sets.**

| **Exercise** | **Execution** |
| --- | --- |
| **Exercise 1: Pelvic lift**  **Starting position:**  Lie on your back with your knees bent and your feet flat on the floor. Keep your arms by your sides with your palms. facing down.  Before you start lifting your hips, engage your abdominal muscles. Press through your heels and tense your glutes as you lift your hips off the ground. Your body should form a straight line from your shoulders to your knees when you're at the top of the movement. Hold up to 5 seconds. Slowly lower your hips back to the starting position.  Hold for up to 5 seconds. **Number of repetitions:** **12; 3 sets** | 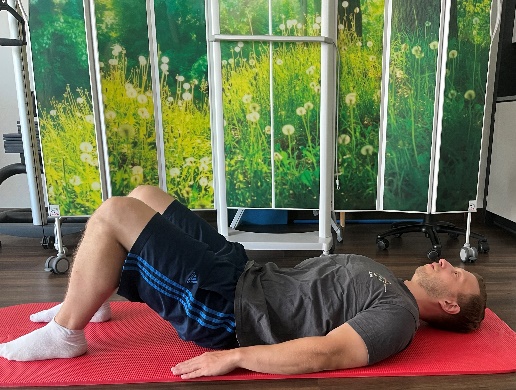    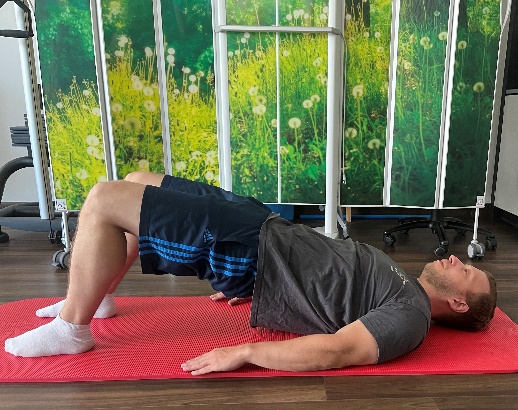 |
| **Exercise 2: Quadruped stand**  **Starting position:**  Kneel on the floor. Support yourself on the yoga mat with the palms of your hands. Tense your abdomen and elongate your spineour back. Now lift the right leg and the left arm. The leg is extended. The thumb points upwards.  Hold this position for 10 seconds and now lower the arm and leg again. **Number of repetitions:** **12; 3 sets** | 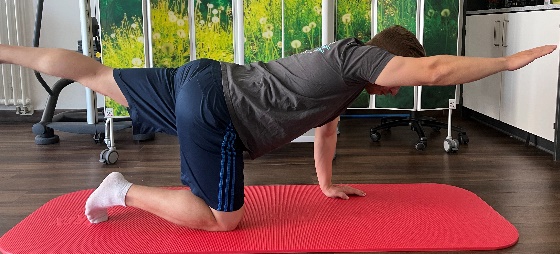 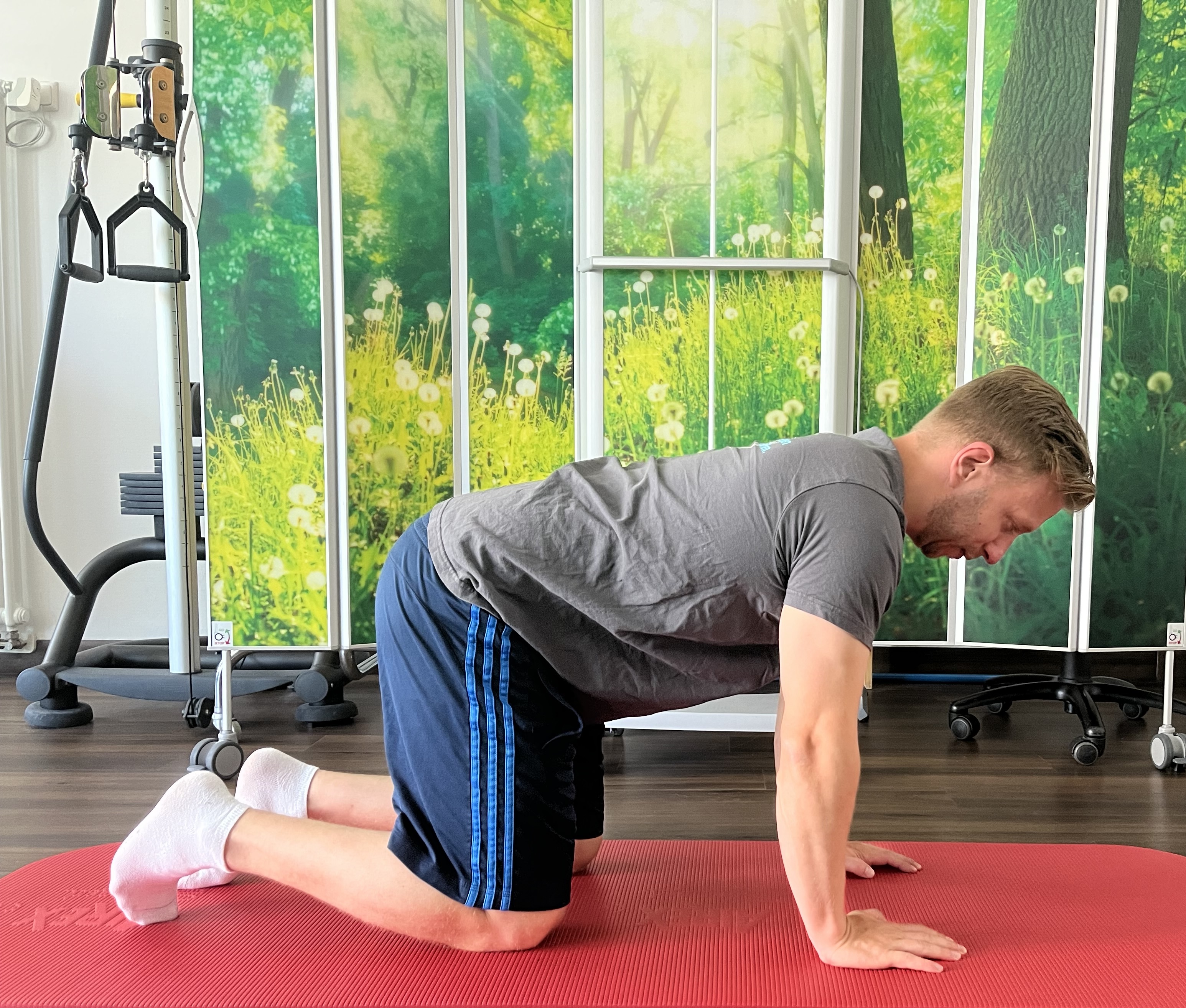 |
| **Exercise 3: Plank**  **Starting position:**  Knees remain on the floor, only the hips and upper body lift off the floor.  Put tension on the abdominal muscles and buttocks. **Hold 30-45 seconds; 2 Repetitions**  **Variation:** If this exercise is too easy over time, lift the knees and hips so that the body becomes a line. | 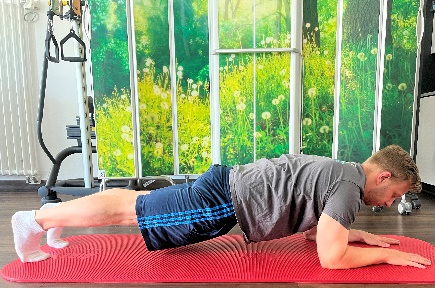 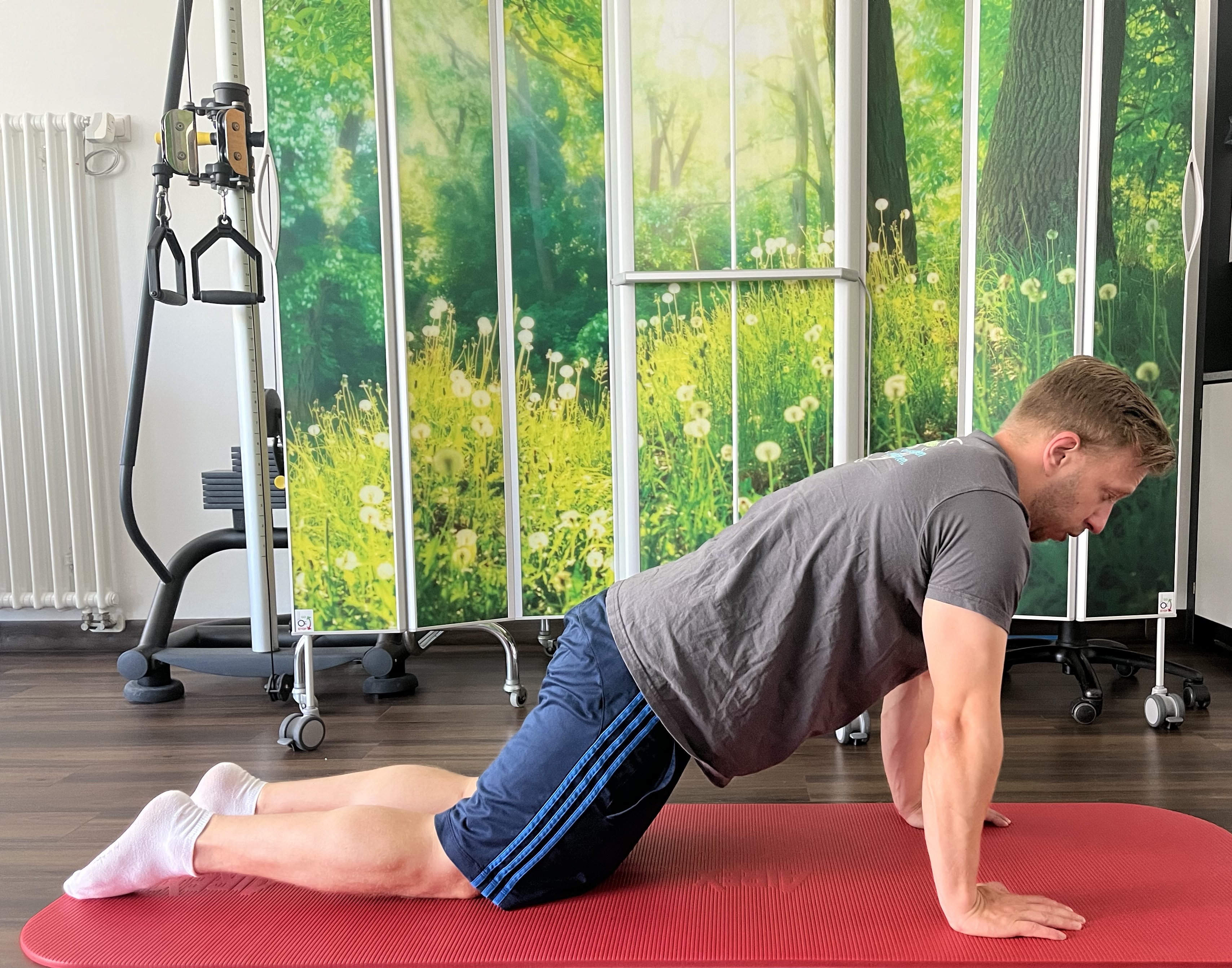 |
| **Exercise 4: Push-ups with knees on the floor:**  Knees remain on the floor, the upper body lowers towards the floor and is pushed up by the arms.  **Number of repetitions**: **8-12; 3 sets**  **Variation:** If the position is too difficult or you have problems performing it, you can also perform it on the wall. Depending at what angle your feet/legs are, the difficulty can be controlled. | 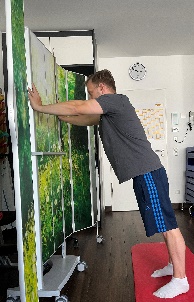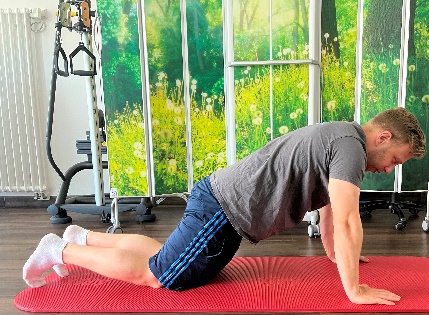 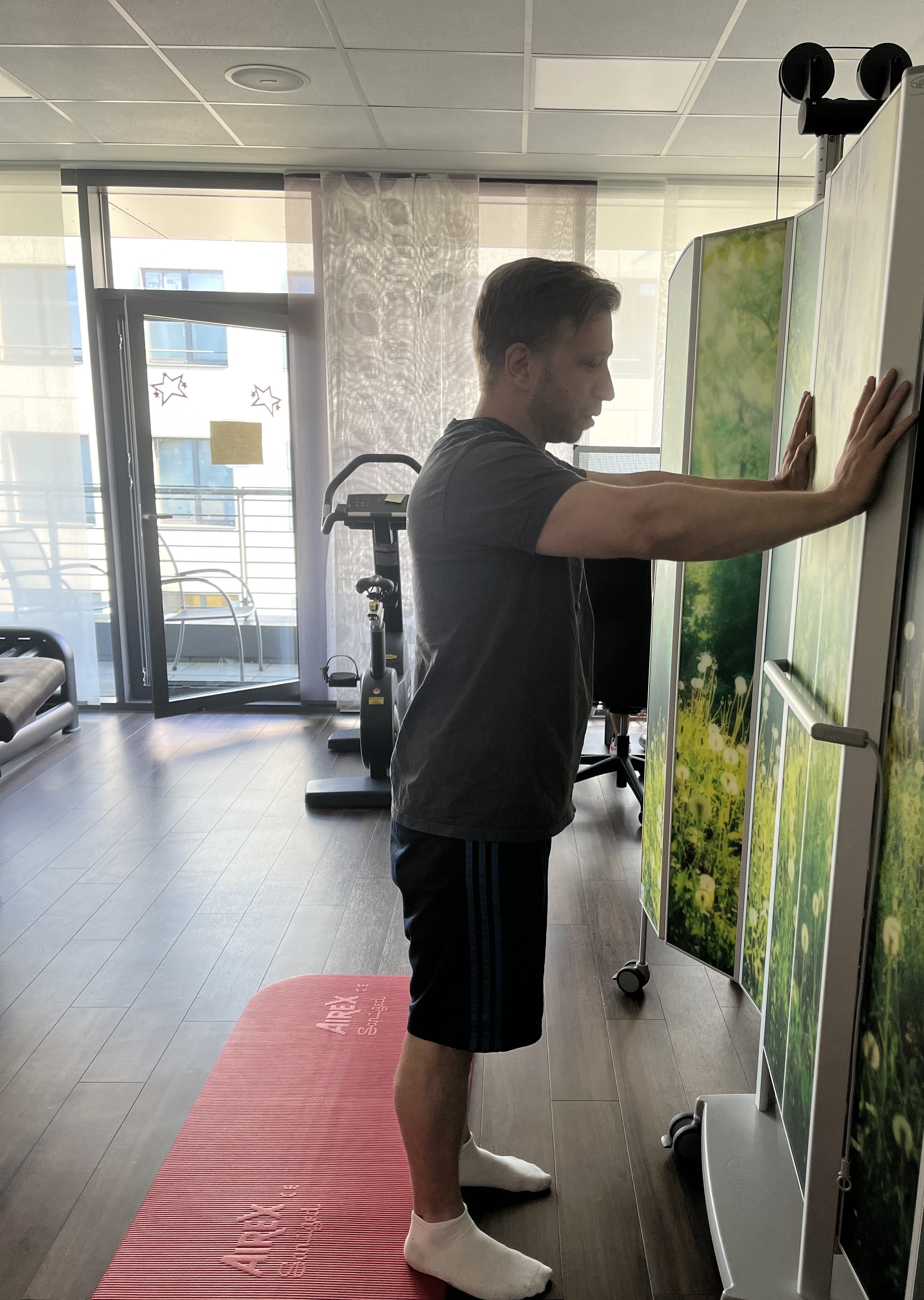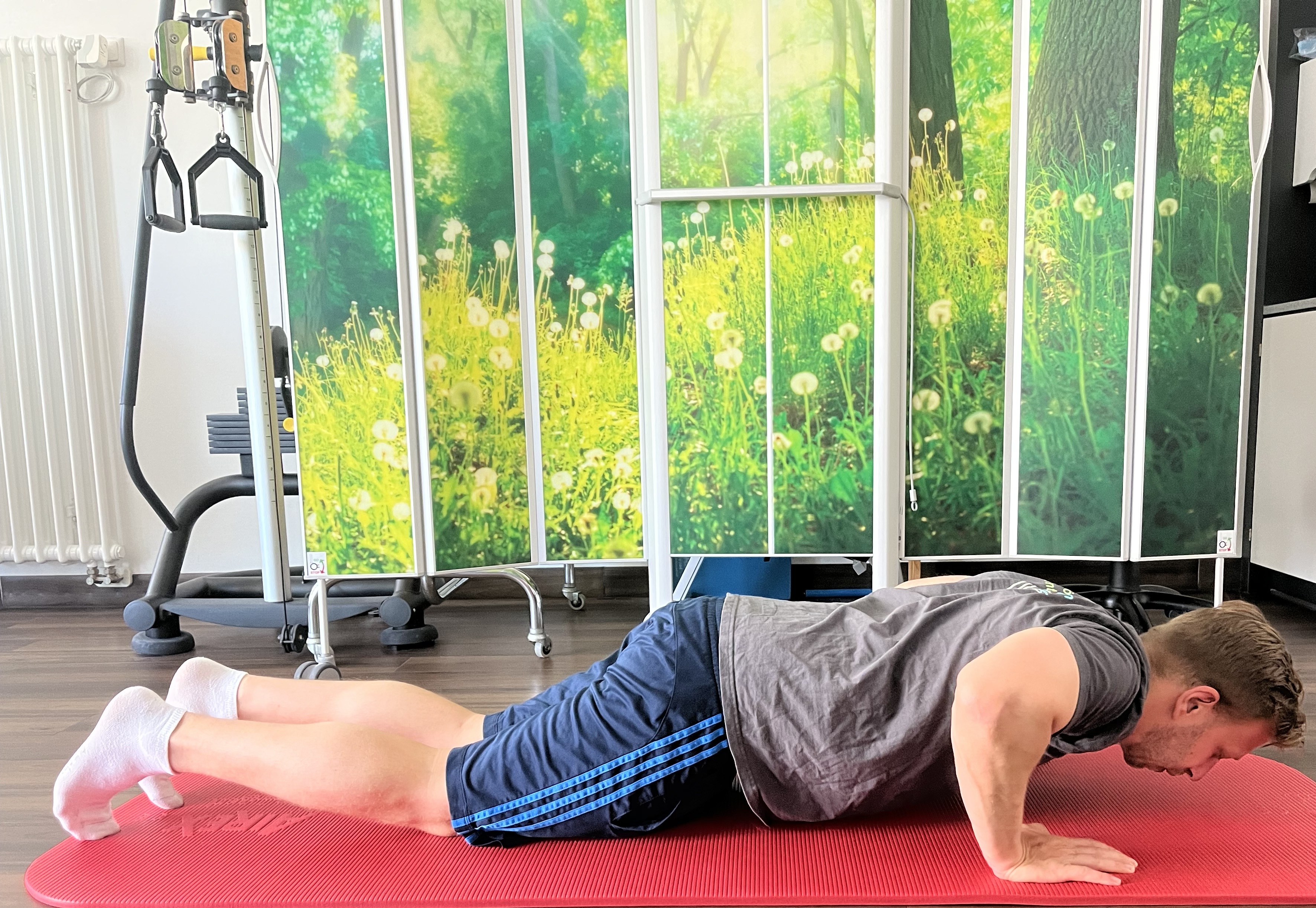 |
| **Exercise 5: Shoulder pull**  Stand upright with legs shoulder-width apart**.** The resistance band is under the feet. Pull your arms up to the sides at shoulder level, holding onto the resistance band.  Hold at the maximum position for five seconds and slowly go back down so that the arms are at the sides of the body.  Number of repetitions: **12; 3 sets** | 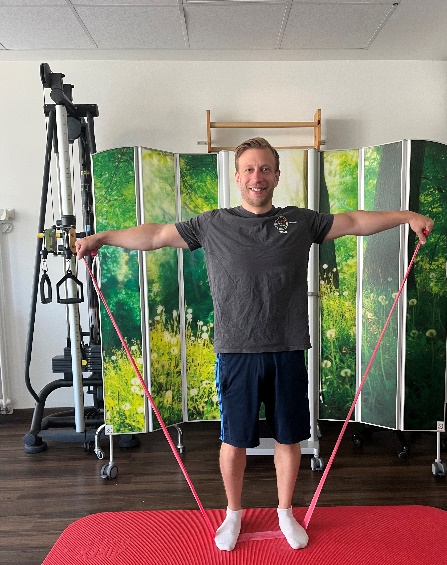 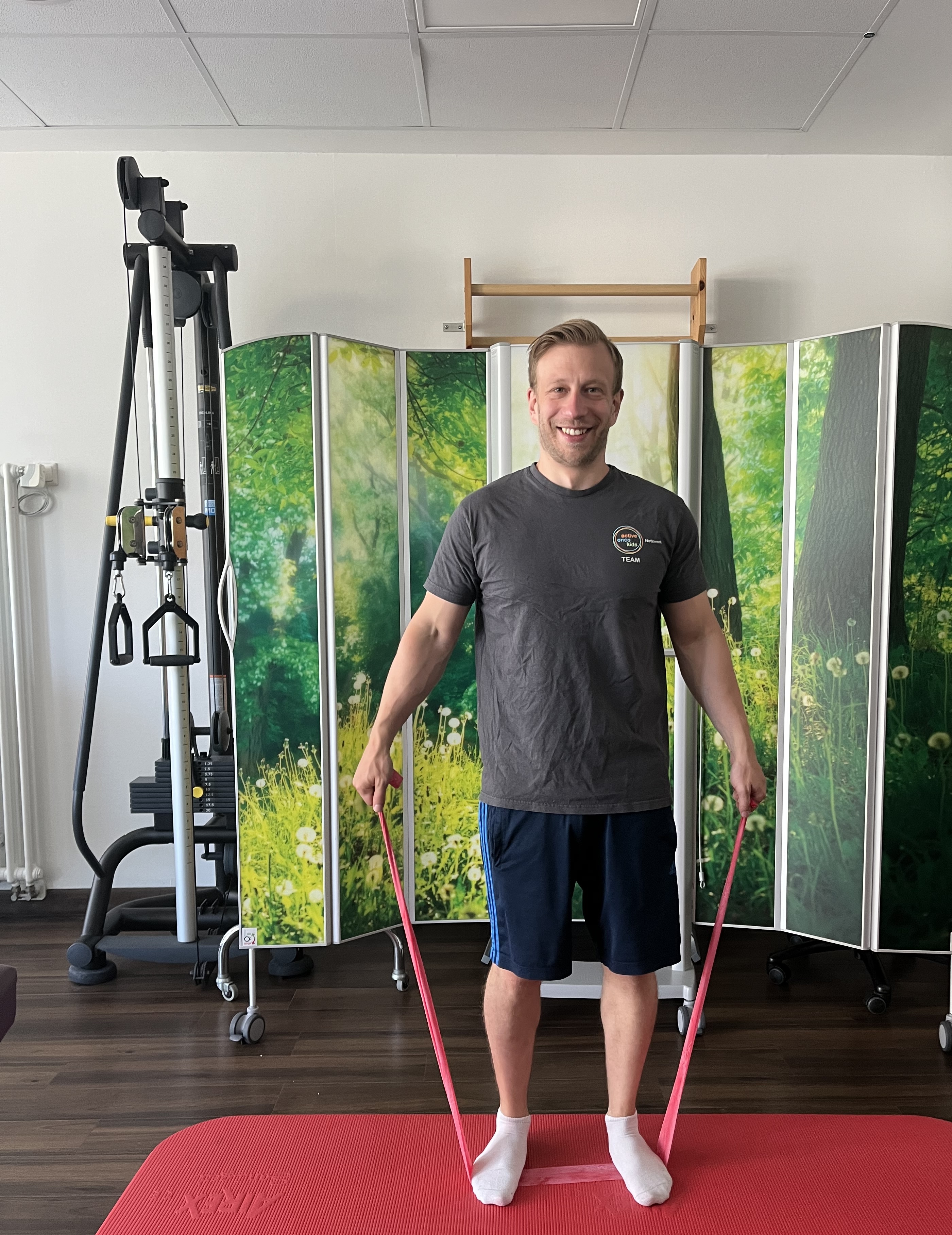 |
| **Exercise 6: Stand up and sit down**  Starting position:  While seated, feet close to chair, arms above shoulder height, stand up and sit down.  Feet are hip-width and slightly oblique. Both knees point slightly outward. If you are unsure about standing up, you can hold on to another chair/rail to have an external help.  If you cant hold up your arm anymore just keep them in front of youre chest.  **Number of repetitions:** **12; 3 sets** | 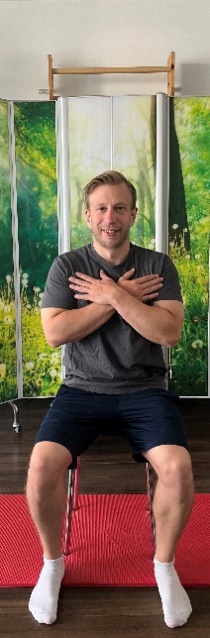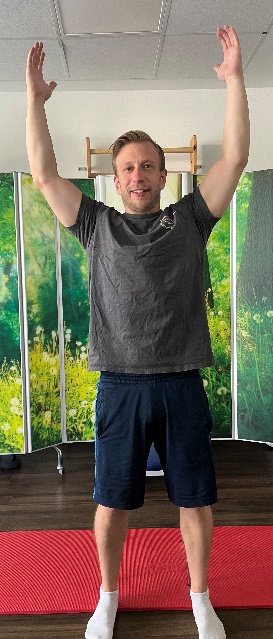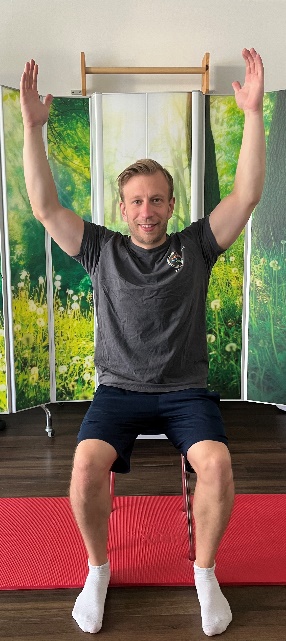 |
| **Exercise 7: Pulling Exercise**  Starting position: sitting or standing  Upper body slightly forward, straight back  Arms stretched out in front of the body  While the upper body does not move, simultaneously bring the elbows back along the body, hold briefly at hip level and then slowly return.  **Number of repetitions:** **12; 3 sets** | 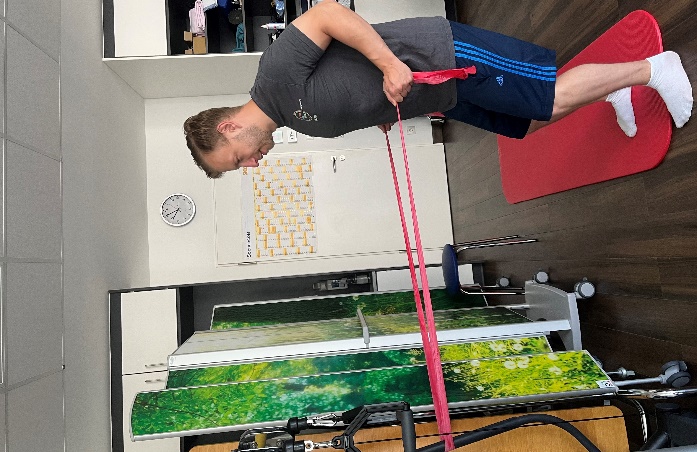 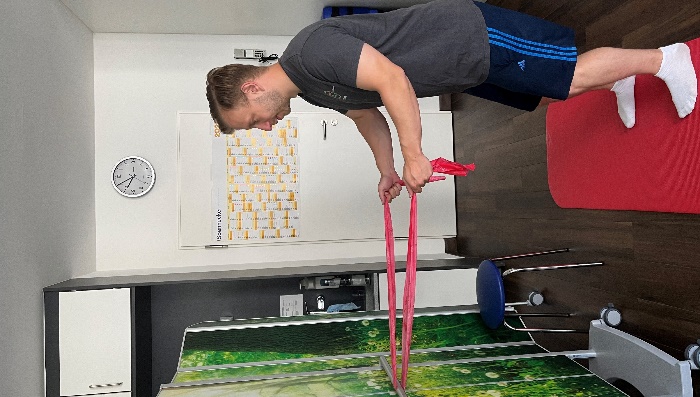 |
| **Exercise 8: Sidesteps**  Starting position:  Stand shoulder-width apart in a slight bend of the knees, upper body slightly bent forward.  Wrap the resistance band around both thighs above the knee.  Take small steps sideways.  **Exercise can also be done lying down!**  **Number of repetitions:** **12; 3 sets** | 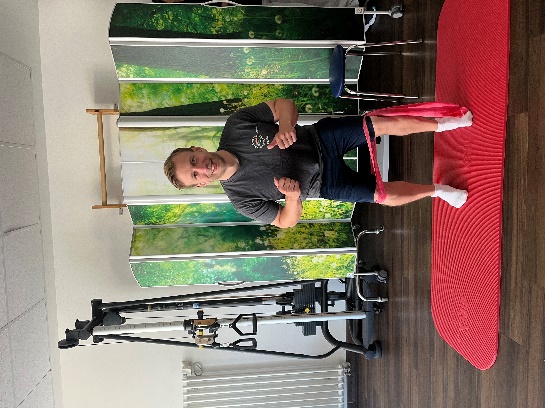 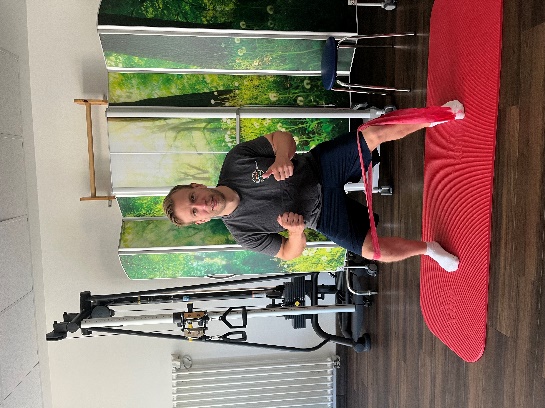 |

**Exercise catalog Cool-Down approx. 5-10 minutes-**

**Breathing exercises**

| **Exercise** | **Execution** |
| --- | --- |
| Deep Breathing | Lie with the back on the floor/mat, hands in front of the chest. Exhale through the mouth, inhale through the nose. When exhaling press the fingers against each other. |
| Draw breath | In a hip-width stance with arms hanging loosely down in front of the abdomen, form a kind of "trowel". On the inhale the hands go up, on the exhale the hands turn and press down. |
| Breathing window | Lie with your back on the mat and bend your legs slightly. The hands clasp each other at the wrist and rest on the chest. On the inhale the hands go over the head and on the exhale they come back to the chest. |
