# Supplementary material for: P-move: a randomized control trial of exercise in patients with advanced pancreatic or biliary tract cancer (aPBC) receiving beyond first-line chemotherapy
Source: Support Care Cancer. 2024 Jun 15;32(7):437. doi: 10.1007/s00520-024-08650-9 (PMC11180022; doi:10.1007/s00520-024-08650-9)
Supplement: Supplementary file 2 — (DOCX 15 kb) [file 520_2024_8650_MOESM2_ESM.docx]

**Recommendations on physical activity and exercise in a cancer trajectory**

Exercise and an active lifestyle lead to positive effects on the patient's well-being during cancer therapy. Patients with advanced cancer show improvements in endurance and strength performance, physical fitness, health-related quality of life, and tumor-associated fatigue symptoms through increased physical activity (1-2). Regular physical activity is also considered as preventive measure e.g. cardiovascular disease and other conditions.

**The World Health Organization recommends for an adult person (3):**

- At least 150 minutes of physical activity at a moderate intensity or 75 minutes of vigorous activity per week.
- For added benefit, physical activity should be increased to 300 minutes of moderate intensity activity
- In addition, strength training of the major muscle groups should be performed two days a week

The American Cancer Society and Exercise and Sport Science Australia (ESSA) conclude, based on numerous studies, that sport and exercise in cancer result in reduced mortality, improved functionality and quality of life. Both associations recommend physical activity during disease and treatment (4-5).

| **Examples of moderate intensity** | **Examples of intense intensity** |
| --- | --- |
| - Walking or going for a stroll | - Jogging, running |
| - Light gymnastic/stretching exercises | - Gymnastics |
| - Light gardening | - Heavy gardening (e.g. chopping wood) |
| - Light swimming | - Running sports (soccer, basketball, etc.) |
| - Slow cycling | - Fast swimming and cycling |

In principle, it is still possible to talk during moderate intensity. In the case of intensive sporting activities, conversation is no longer possible without breathing in between.

From preliminary studies at the West German Tumor Center, we know that many patients with advanced tumor disease have to overcome various barriers in order to be physical active. Especially at the time of tumor therapy, many patients feel tired, weak or have other symptoms such as nausea, pain or shortness of breath. Despite any tumor-related symptoms, you should try to move as much as you can. Every movement counts to increase your weekly activity level!

Dittus, K. L., Gramling, R. E., & Ades, P. A. (2017). Exercise interventions for individuals with advanced cancer: a systematic review. Preventive medicine, 104, 124-132.

Schwartz, A., de Heer, H. D., & Bea, J. W. (2017). Initiating exercise interventions to promote wellness in cancer patients and survivors. Oncology (Williston Park, NY), 31(10), 711.

https://www.who.int/news-room/fact-sheets/detail/physical-activity

4 Schmitz, K. H., Courneya, K. S., Matthews, C., Demark-Wahnefried, W., Galvão, D. A., Pinto, B. M., ... & Schneider, C. M. (2010). American College of Sports Medicine roundtable on exercise guidelines for cancer survivors. Medicine & Science in Sports & Exercise, 42(7), 1409-1426.

Hayes, S. C., Newton, R. U., Spence, R. R., & Galvão, D. A. (2019). The Exercise and Sports Science Australia position statement: Exercise medicine in cancer management. Journal of science and medicine in sport.

Frikkel, J., Götte, M., Beckmann, M., Kasper, S., Hense, J., Teufel, M., ... & Tewes, M. (2020). Fatigue, barriers to physical activity and predictors for motivation to exercise in advanced Cancer patients. BMC Palliative Care, 19(1), 1-11.
